# Laxative effects of Salecan on normal and two models of experimental constipated mice

**DOI:** 10.1186/1471-230X-13-52

**Published:** 2013-03-20

**Authors:** Mengyi Zhou, Ping Jia, Jinping Chen, Aihui Xiu, Yue Zhao, Yibei Zhan, Peng Chen, Jianfa Zhang

**Affiliations:** 1 Center for Molecular Metabolism, Nanjing University of Science & Technology, 200 Xiaolingwei Street, Nanjing, 210094, China

**Keywords:** Salecan, Constipation, Intestinal motility, Loperamide, Clonidine

## Abstract

**Background:**

Constipation is one of the most common gastrointestinal complaints with a highly prevalent and often chronic functional gastrointestinal disorder affecting health-related quality of life. The aim of the present study was to evaluate the effects of Salecan on fecal output and small intestinal transit in normal and two models of drug-induced constipation mice.

**Methods:**

ICR mice were administrated intragastrically (i.g.) by gavage with 100, 200 and 300 mg/kg body weight (BW) of Salecan while the control mice were received saline. The constipated mice were induced by two types of drugs, loperamide (5 mg/kg BW, i.g.) and clonidine (200 μg/kg BW, i.g.), after Salecan treatment while the control mice were received saline. Number, weight and water content of feces were subsequently measured. Small intestinal transit was monitored by phenol red marker meal.

**Results:**

Salecan (300 mg/kg BW) significantly increased the number and weight of feces in normal mice. In two models of drug-induced constipation, Salecan dose-dependently restored the fecal number and fecal weight. The water content of feces was markedly affected by loperamide, but not by clonidine. Treatment with Salecan significantly raised the fecal water content in loperamide-induced constipation mice. Moreover, Salecan markedly stimulated the small intestinal transit in both loperamide- and clonidine-induced constipation model mice.

**Conclusions:**

These results suggest that Salecan has a potential to be used as a hydrophilic laxative for constipation.

## Background

Constipation, defined as infrequent or difficult evacuation of feces [1], is a worldwide functional gastrointestinal disorder. A systematic review recorded prevalence rates in six different population groups from Asia ranging widely from 11.6% to 29.6% [2]. In general, constipation appears to be more common in the elderly, women, nonwhites, and persons in lower socio-economic and education classes [3]. Constipation also significantly impacts health-related quality of life in constipated people [4]. A primary mechanism for slow-transit constipation is a failure of peristalsis to move luminal contents through the colon results in more time for bacterial degradation of stool solids and more time for salt and water absorption, thus reducing stool frequency and stool weight dramatically [5]. Medical therapy for constipation contains traditional laxatives and agents. The former can induce defecation or modify stool consistency to make defecation easier, while the latter targets presumed defects in colonic neuromuscular function [5,6]. Although a common problem, the treatment of constipation has been far from satisfactory [7].

Beta-glucans, naturally occurring polysaccharides with poly-branched beta-(1→3)-D-glucans or beta-(1→6)-D-glucose side chains, are a major component of the bran of cereal plant and the cell wall of bacteria and fungi [8,9]. A number of studies demonstrate beta-glucans may be beneficial in gastrointestinal disease prevention and health promotion, including reduction of cholesterol absorption and bile acids excretion [10], fermentation by intestinal bacteria that yields short chain fatty acids [11], resistance to enteric bacterial and viral infections [12,13], stimulation autochthonous *Lactobacillus* populations in the colon [14], and prevention of colorectal cancer by effecting immune and cancer cells [15]. In addition, early observers suggest that a decreasing prevalence of constipation is associated with an increasing dietary fiber intake, depending on their ability to avoid digestion and absorption in small intestine and to escape bacterial metabolism in colon [16]. Cummings [17] has tabulated the efficacy of different fibers in terms of increased fecal weight per gram of administered fiber, including wheat bran, psyllium, cellulose, oats, corn, legumes, and pectin. Psyllium polysaccharide from *Plantago ovate* is approved by the FDA for its proven laxative effects as available fiber supplements [18,19].

Salecan, produced by *Agrobacterium* sp. ZX09, is a novel high molecular, water-soluble extracellular polysaccharide, and its structure is proven to consist of the following repeating unit:→3)-β-D-Glcp-(1→3)-[ β-D-Glcp-(1→3)-β-D-Glcp-(1→3)]3-α-D-Glcp-(1→3)-α-D-Glcp-(1→ [20]. As a new beta-glucan with special molecular structure, its safety has been demonstrated in the acute and subchronic experiment [21]. Supplementation with Salecan reduced adiposity and improved glucose tolerance in high-fat diet-fed mice through disturbing bile acid-promoted emulsification in intestine [22]. Its rheology study indicates that Salecan has a non-Newtonian viscosity behavior, and could be utilized in the food industry [23]. In the present study, we estimated the laxative effects of Salecan on fecal output and small intestinal transit in normal and two models of drug-induced constipation mice. Our results suggest that Salecan may be used as a hydrophilic laxative for constipation.

## Methods

### Salecan

Salecan was prepared according to previous methods [20]. Briefly, *Agrobacterium* sp. ZX09 used in this study was isolated from a soil sample from the ocean coast of Shandong, China. Cultures were maintained on Htm agar containing NaH_2_PO_4_ (1 g) , KNO_3_ (3 g), CaCl_2_ (0.07 g), MgCl_2_ (0.2 g), FeSO_4_·7H_2_O (0.0125 g), MnSO_4_ (0.003 g), ZnCl_2_ (0.0075 g), sucrose (20 g), agar (9 g) and H_2_O (1000 ml), pH 7.2. A colony of the strain ZX09 was inoculated into a 250 ml flask containing 50 ml medium consisting of 2 % sucrose and mineral salt solution. The inoculated preparation was incubated at 28°C on a rotary shaker at 220 rpm for 24 h. A 0.5 ml portion was transferred to a 250 ml flask containing 50 ml fermentation medium. Fermentation was performed on a rotary shaker at 220 rpm for 48 h. The culture broth was diluted more than 3 times with de-ionized water and centrifuged at 12 000 × *g* for 30 min to separate cells from the supernatant. The supernatant was added to two volumes of 95 % ethanol. Productivity of Salecan was expressed in terms of the weight after ethanol precipitation collected by centrifugation at 6000 × *g* for 15 min and dried under reduced pressure. The chemical composition, solution viscosity and water holding capacity (WHC) of Salecan were shown in Table 1.

**Table 1:** The chemical composition and physical characteristics of Salecan

**Characteristic**	**Value**
Composition	
Sugar	77.13%
Protein	6.2%
Moisture	5.2%
Ash	10.28%
Viscosity	
0.4 g/60 ml H_2_0	3260 mPa·s
0.8 g/60 ml H_2_0	7850 mPa·s
1.2 g/60 ml H_2_0	9500 mPa·s
Water holding capacity	9.7 g/g H_2_0

### Animals

Male ICR mice (8 weeks old) were purchased from SLAC (Shanghai Laboratory Animal Center, Shanghai). All animals, used after 1 week of acclimation (temperature 22 ± 2°C; 12/12 h light/dark cycles), had free access to standard lab chow and water. All animal procedures were approved by the Institutional Animal Care and Use Committee of Nanjing University of Science and Technology.

### Fecal parameters measurements in normal mice

Mice, given food and water *ad lib*, were randomly divided into four groups (n = 5 in each group): control and Salecan (three groups of different dosage). After either Salecan (100, 200 and 300 mg/kg BW, doses based on Maeda et al. [24] and Shan et al. [25]) or saline was administered (i.g.), the animals were immediately placed in clean transparent cages individually and allowed access to their standard lab chow and top water *ad libitum*. Then, feces for each mouse were collected, counted and weighted at 0–8 h period. The number and weight of feces were expressed in terms of the total number and wet weight per mouse.

### Fecal parameters measurements in two types of constipation model mice

The number and weight of feces for each mouse in loperamide- and clonidine-induced constipation model mice were measured as the method of Kakino et al. [26,27]. The mice, given food and water *ad lib*, were administered (i.g.) saline or Salecan at 100, 200 or 300 mg/kg BW, and then administered loperamide (5 mg/kg BW, dissolved in 1% Tween 80/saline, i.g.) at 1 h after or clonidine (200 μg/kg BW, dissolved in saline, i.g.) at 45 min after Salecan treatment. Then, the experimental processes were in accordance with Section 2.3. After weighted, the wet feces for each mouse were dried at 105°C for 48 h. The water content of feces was calculated as:fecal water content (%) = (feces weight before dried - feces weight after dried)/feces weight before dried × 100.

### Determination of small intestinal transit

Small intestinal transit (SIT) was determined using a phenol red marker meal (0.5 ‰ phenol red indicator in 1.5 % methylcellulose; 10 ml/kg BW, i.g.), followed the previous studies [26]. Administering of three doses of Salecan and two models of constipation were in accordance with Section 2.4. After fasted for 14 h with water *ad libitum*, all mice were administered by i.g. with either Salecan or saline at 1 h before loperamide or at 45 min before clonidine. After 30 min, a phenol red marker meal was administered to experimental mice. After 20 min, the mice were sacrificed by cervical dislocation under anesthesia with diethyl ether. The small intestine from the pylorus to the blind intestine was carefully removed. The SIT (%) for each animal was calculated as: distance traveled by phenol red marker meal/total length of the small intestine × 100.

### Statistics

All data are expressed as mean ± SEM. Differences between groups were analyzed by one-way analysis of variance (ANOVA) followed by the Tukey test. Differences between means were considered statistically significant at *p* < 0.05.

## Results

### Increased fecal output in normal mice treated with Salecan

**Figure 1:**
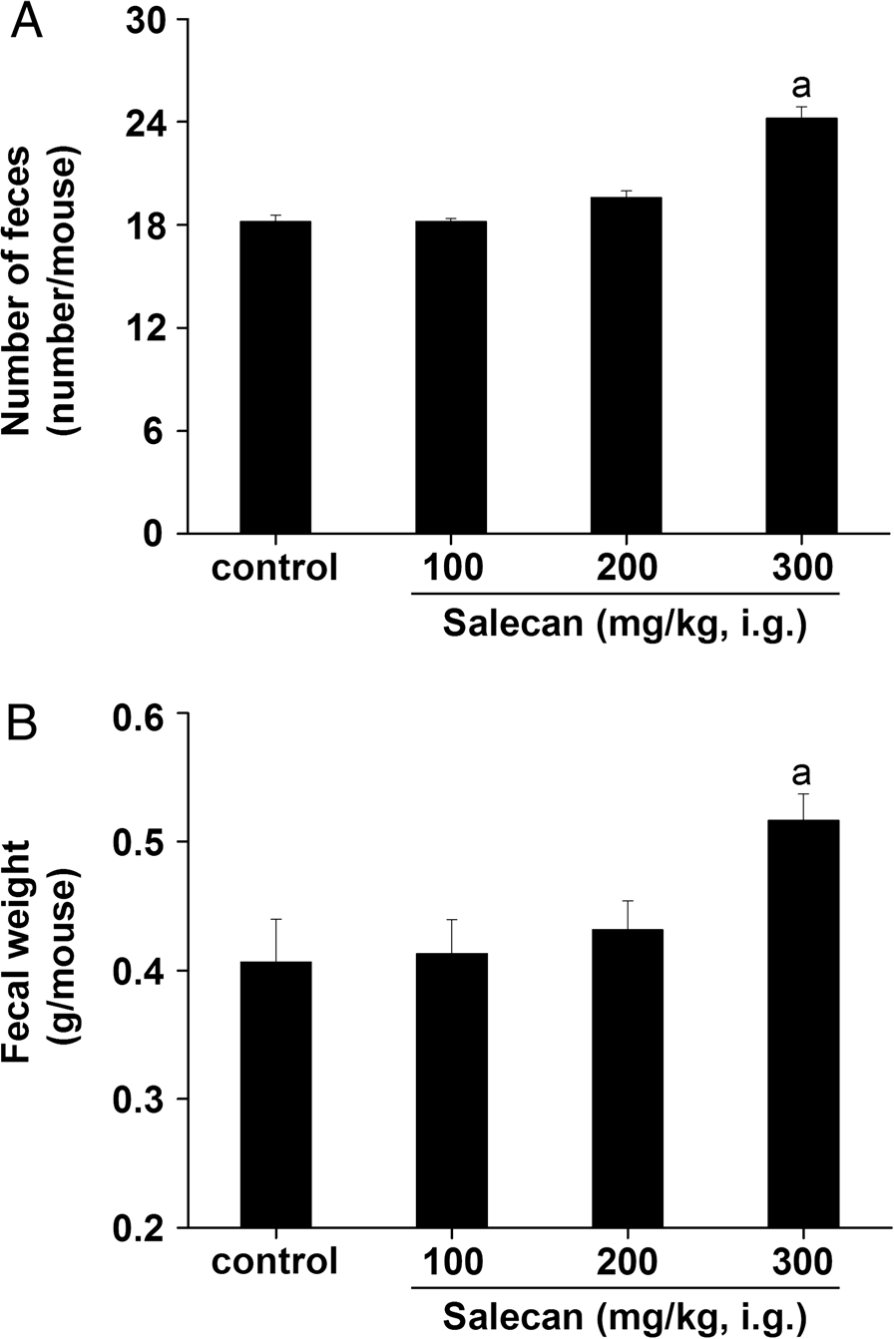
**Effects of Salecan on number (A) and weight (B) of feces in normal mice.** All values are presented as mean ± SEM (n = 5). ^a^*p* < 0.05 compared with control.

The effects of Salecan on number and weight of feces in normal mice were shown in Figure 1. Compared with the control, treatment with Salecan (300 mg/kg BW) produced a significant increase in number of feces (Figure 1A) and fecal weight (Figure 1B).

### Salecan improved fecal output character in two types of constipation mice

**Figure 2:**
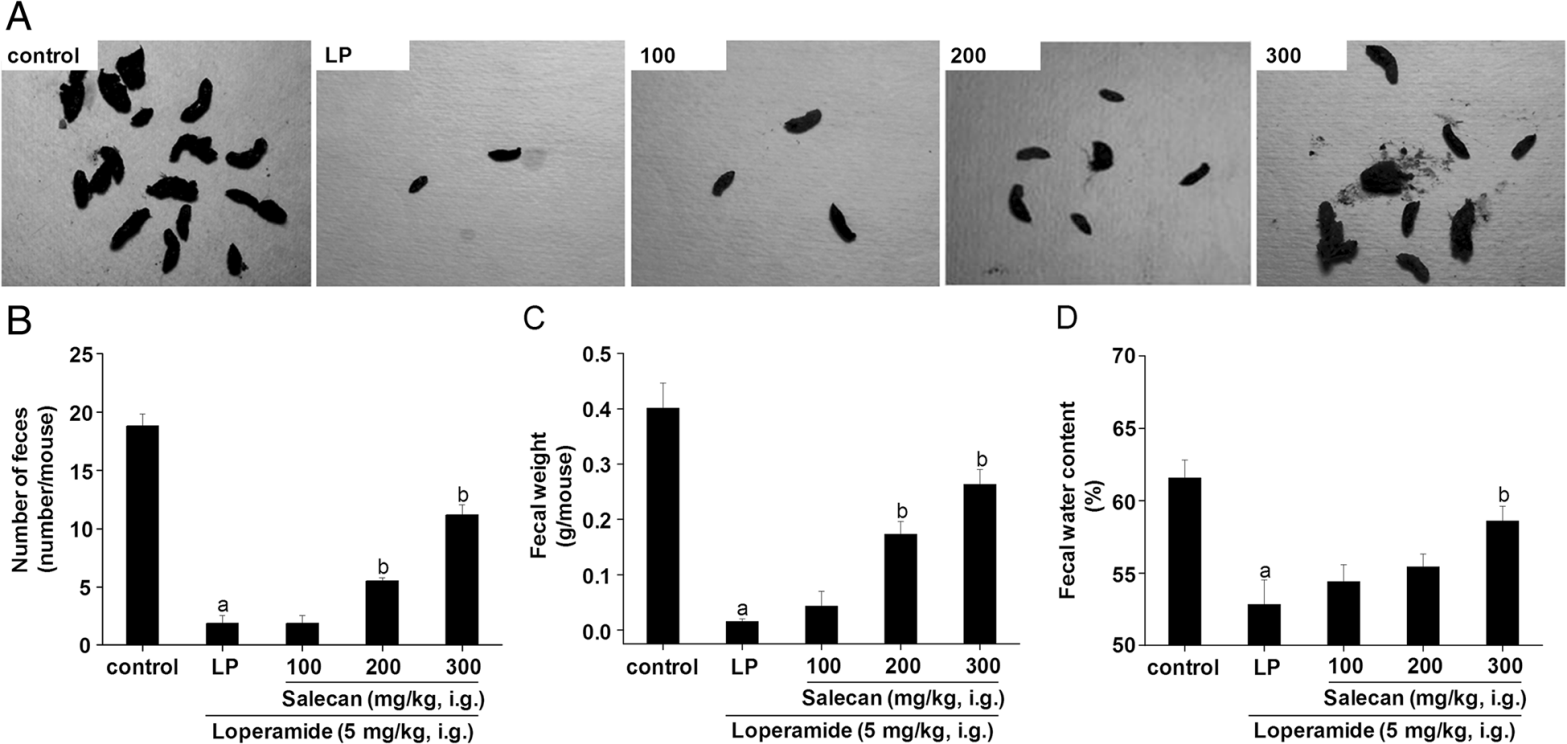
**Effects of Salecan on number (A, B), weight (C) and water content (D) of feces in loperamide-induced constipation mice.** All values are presented as mean ± SEM (n = 8). ^a^*p* < 0.05 compared with control; ^b^*p* < 0.05 compared with LP. LP, loperamide-treated group as constipated control.

A model of spastic constipation was induced by loperamide. Loperamide obviously reduced number, weight and water content of feces (Figure 2). Salecan counteracted the decrease in number and weight of feces induced by loperamide (Figure 2A, B, C). The effect was significant starting from the 200 mg/kg BW oral dose. There was a significant restoration of fecal water content in Salecan group at 300 mg/kg (95.14 ± 1.68% of control) compared with the LP group (85.81 ± 2.77% of control) (Figure 2D).

**Figure 3:**
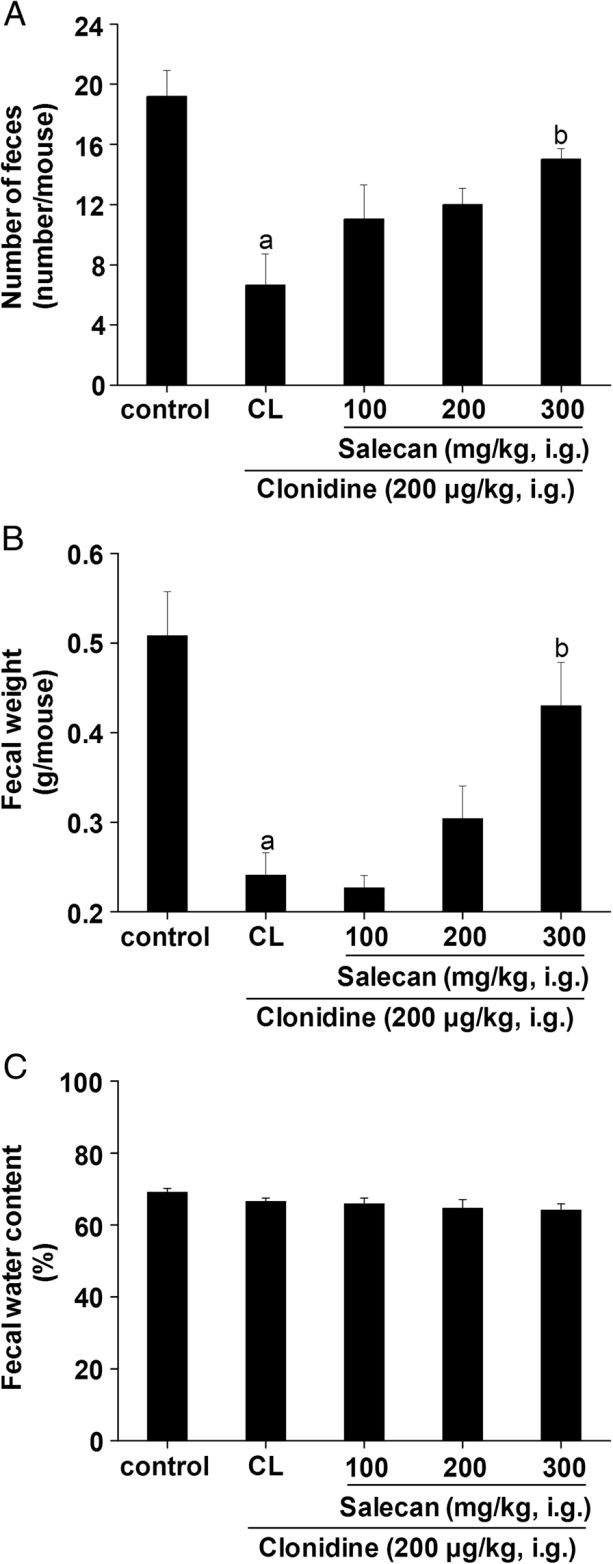
**Effects of Salecan on number (A), weight (B) and water content (C) of feces in clonidine-induced constipation mice.** All values are presented as mean ± SEM (n = 8). ^a^*p* < 0.05 compared with control; ^b^*p* < 0.05 compared with CL. CL, clonidine-treated group as constipated control.

A model of atonic constipation was induced by clonidine. Clonidine significantly reduced the fecal number from ~19 to ~7 and fecal weight from ~0.5 g to ~0.24 g (Figure 3A, B). Salecan at a dose of 300 mg/kg obviously increased the fecal number and fecal weight to ~15 and ~0.43 g, respectively (Figure 3A, B). There was no significant difference between the groups in fecal water content (Figure 3C).

### Effects of Salecan on the SIT

**Figure 4:**
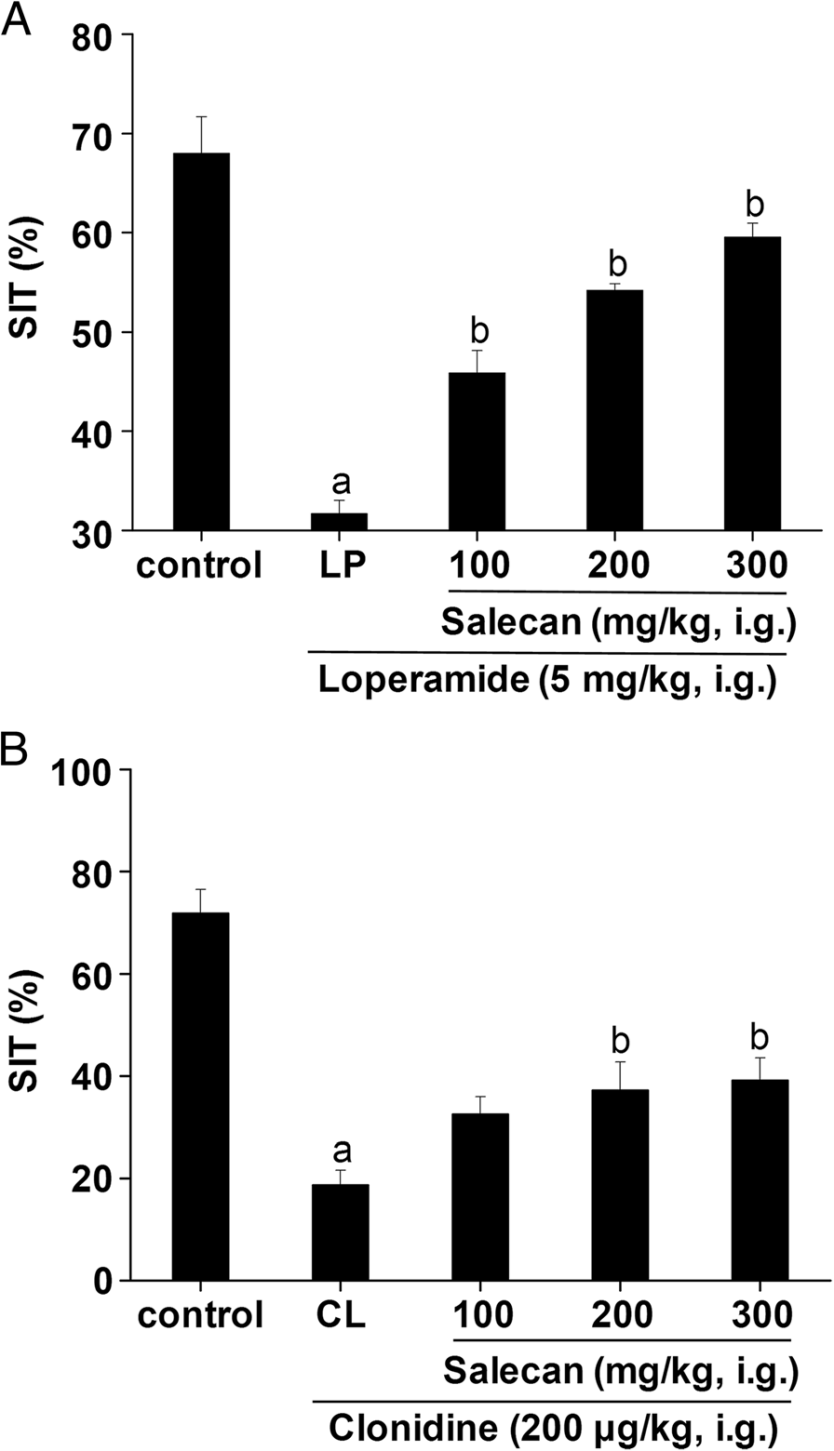
**Effects of Salecan on small intestinal transit in loperamide- (A) and clonidine-induced (B) constipation mice.** All values are presented as mean ± SEM (n = 8). ^a^*p* < 0.05 compared with control; ^b^*p* < 0.05 compared with LP or CL. LP, loperamide-treated group as constipated control; CL, clonidine-treated group as constipated control.

After loperamide or clonidine administration, the SIT were significantly inhibited (Figure 4). The treatment with Salecan increased the SIT in a dose dependent manner in both loperamide- and clonidine-induced constipation model mice (Figure 4).

## Discussion

Constipation is a highly prevalent functional gastrointestinal disorder, affecting the quality of life in constipated persons [7], and the use of dietary fiber in the prevention and treatment of constipation is a common practice in many countries of the world [16,28]. The present study has evaluated laxative effects of Salecan on normal as well as on loperamide- and clonidine-induced constipation mice.

As an agent for functional bowel disorders like diarrhea, loperamide used as constipation inducer is well documented. The drug inhibits intestinal fluid secretion and intestinal motility, leading to delay fecal evacuation time and intestinal luminal transit [29], and is used to induce a model of spastic constipation. On the other hand, clonidine also has a potent antidiarrheal effect, and has a suppressive effect on gastrointestinal contractions [30]. This drug can cause a model of atonic constipation. The observed decrease in the number and weight of feces by the treatment with these two drugs indicated induction of constipation in mice. Similar observation was reported by Kakino et al. [27].

Salecan is an identified extracellular macromolecule from *Agrobacterium* sp. ZX09, mainly composed of β-(1→3)-D-glucosidic linkages as the main backbone structure, together with a small portion of α-(1→3)-D-glucosidic linkages [20]. Salecan is a non-toxic and water-soluble beta-glucan with excellent rheological properties [21,23]. Bacterial extracellular polysaccharides (e.g. dextran) can be easily produced and applied in food industry [31]. Beta-glucans are not digestible due to a lack of the hydrolase in gastrointestinal tract. Furthermore, Salecan increased fecal weight and fecal number in normal mice in present study. Similar observation was reported by Nakamura et al. [32] where brewer’s yeast cell wall, which was composed mainly of polysaccharides, significantly increased fecal frequency and weight in normal rats. Due to these properties, it is presumed that Salecan has favorable effects on prevention of constipation.

The effects of Salecan on constipation in this study were tested in loperamide- and clonidine-induced constipation mice. The administration of Salecan to the constipated mice was effective in increasing the fecal number and fecal weight, which were indications of the laxative character of Salecan. Unlike clonidine, loperamide also markedly decreased the water content of feces through inhibition of intestinal fluid secretion. Treatment with Salecan significantly raised the fecal water content in loperamide-induced constipation mice. And the SIT time was shortened by the treatment with Salecan in both constipation models. The laxative effectiveness of dietary fibers, as bulk agents, depends on their WHC and swelling force [5]. Salecan was proved to have a high WHC. It may thus be concluded that the fecal output character was affected by the WHC and swelling force of Salecan in small intestine. This swelling force serves as a stimulus to defecation. Xu et al. [33] reported that supplementation of the diet with partially defatted flaxseed meal markedly decreased gastrointestinal transit time as well as increased fecal frequency and weight in constipated mice, which was dependent on luminal bulk. In addition, given the fact that soluble fibers may delay nutrients digestion and absorption by absorbing large quantities of water and forming gels in the gastrointestinal tract [34], Salecan might be possible to prevent the absorption of loperamide and clonidine in this results.

## Conclusion

Our results demonstrate that Salecan, as a new structural glucan, alleviates the symptom of loperamide- and clonidine-induced constipation. Because of its easy availability, Salecan could be recommended as a cost-effective alternative for constipation.

## Abbreviations

SIT: Small intestinal transit; WHC: Water holding capacity

## Competing interests

The authors declared no conflict of interest.

## Authors’ contributions

MYZ substantially contributed to study conception and design, as well as acquisition, analysis, and interpretation of data, drafting and revision of the manuscript. PJ and YBZ participated in the acquisition and analysis of data. AHX conceived of the study conception and design. JPC and YZ was involved in the experiments. PC helped to draft and revise the manuscript. JFZ contributed to study design, drafting and revision of the manuscript, and acquisition of funding. All authors read and approved the final manuscript.

## Pre-publication history

The pre-publication history for this paper can be accessed here:

http://www.biomedcentral.com/1471-230X/13/52/prepub
